# A Spline Curve Fitting Model for Towed Streamer Positioning in Marine Seismic Exploration

**DOI:** 10.3390/s25165114

**Published:** 2025-08-18

**Authors:** Haonan Zhang, Kaiwei Sang, Baocai Yang, Chufeng Duan, Lingsheng Lv, Cuilin Kuang, Heng Liu

**Affiliations:** 1 School of Geosciences and Info-Physics, Central South University, Changsha 410083, China; zhanghn7@cosl.com.cn (H.Z.);; 2 Geophysical Division of China Oilfield Services Ltd., Tianjin 300451, China; 3 National Engineering Research Center of Offshore Oil and Gas Exploration, Beijing 100028, China; 4 School of Electronics and Communication Engineering, Sun Yat-sen University, Shenzhen 518107, China

**Keywords:** towed streamer seismic exploration, hydrophone positioning, spline curve model, polynomial curve model

## Abstract

The shape and position information of towed streamers is crucial for both implementing marine seismic exploration operations and analyzing exploration data. Streamer positioning accuracy directly impacts the quality and reliability of seismic imaging. Existing polynomial curve models exhibit deviations between the calculated and actual shapes during streamer turning. This paper proposes a segmented fitting positioning model based on spline curves. It is mathematically rigorous and applicable to complex scenarios. First, the specific function expression of the spline curve model is constructed. Then, using a cubic spline as an example, the segmented fitting method is explained, incorporating smoothness constraints at the connection points. The error equations for positioning observations and the calculation processes for curve parameters and hydrophone coordinates are derived. Finally, the model is verified through simulations and field tests. The experimental results show that, compared with the polynomial curve model, the spline curve model improves positioning accuracy by 47.1% in simulations involving six streamers and by 20.0% and 35.0% in field tests with six and ten streamers, respectively. In straight scenarios, both models perform similarly. Thus, the spline model can effectively reduce the modeling errors of the polynomial curve model under high-curvature conditions.

## 1. Introduction

With significant advantages like high cost-effectiveness and strong adaptability to complex structures, towed streamer seismic exploration is now a mainstream offshore oil exploration method. In actual operations, seismic survey vessels tow single or multiple gun arrays and multiple streamers. According to the planning of the survey line in the work area, streamer operation mainly involves two phases, straight-line towing and offline turning, which alternate during the operation. During straight towing, the vessel collects seismic data along the predetermined survey line. Once the seismic source reaches the designated position, it generates seismic waves. These waves are reflected back from different seabed strata and received by hydrophones on the streamer. Finally, the seismic data are analyzed and interpreted, offering crucial reference information for oil and gas development [1,2]. In this process, the hydrophone positions at the moment of shooting are essential for seismic data analysis. Their positioning accuracy directly impacts the imaging quality of the seismic data and the reliability of the results, thus ensuring the effectiveness of the entire exploration process. Offline turning and line changing are necessary for transitioning between survey lines. After completing the seismic acquisition of the current line, the vessel needs to turn to a new survey line. This process usually involves a 180° turn, which often lasts for several hours. During this stage, situations such as towed streamer entanglement are likely to occur, making it difficult for operators to accurately judge the underwater movement of the streamer and increasing operational risk. Accurate positioning of the towed streamer ensures alignment between the calculated and actual positions. This enables timely and precise adjustments to the streamer position, ensures the efficiency of acquisition operations, and prevents operational delays or data acquisition errors from positioning deviations. In summary, accurate towed streamer position information is vital for efficient operations and risk avoidance.

In the early stages of geometric positioning model development, operations primarily involved a single streamer equipped with only a magnetic compass. Single-streamer analytical positioning models were mainly used. These models, which rely on the relationship between compass azimuth and coordinates, employ methods such as polynomial curves, circular arcs, and coordinate integration to model the towed streamer and determine its shape and the position of the hydrophones [3,4,5,6]. This analytical method, supported by a rigorous mathematical model, determines the streamer shape by solving model parameters and directly or indirectly derives the coordinates of any point on the streamer. Its advantages are that the number of parameters to be solved is relatively small, the method itself is intuitive and easy to apply in practice, and it conforms to the physical characteristics of a smooth streamer shape. However, this approach, which only models based on compass bearings, struggles to fully account for multi-streamer scenarios [7,8,9,10]. At the same time, some physical models and geometric–physical hybrid models have been developed, but they are difficult to apply when only geometric observations are available from the sensors [11,12]. As exploration technology has advanced and demands for towed streamer positioning accuracy and reliability have grown, GPS buoys and acoustic sensors have gradually been deployed on towed streamers. Against this background, in order to better adapt to multi-streamer operations, some scholars have conducted in-depth research and proposed a series of new towed streamer positioning methods. At this stage, the multi-streamer numerical positioning method combining network adjustment and coordinate integration has become the most representative solution [12,13,14,15]. Specifically, coordinate integration is first performed on streamer nodes using compass bearing observations. Then, based on the pre-processed approximate streamer shape, triangulateration network adjustments are carried out. Finally, using the points with high accuracy after adjustment as the reference, each streamer segment is rotated, translated, scaled, and otherwise adjusted to improve the positioning accuracy of the hydrophones on the towed streamer. For example, assuming the basic composition of the towed streamer is a circular arc, polynomial curve, or piecewise linear segment, methods such as arc segments, polynomials, and coordinate integration are used for modeling [16]. In other methods, acoustic node coordinates are obtained using acoustic network adjustment, and then polynomial curve fitting or interpolation is performed on the acoustic nodes to determine the shape of the towed streamer and the coordinates of the hydrophone [17,18]. Yao et al. proposed a multi-step method for determining hydrophone coordinates in 2010, including constrained network adjustment, curve integration, quasi-stable adjustment, and streamer shape adjustment [19]. Yi added virtual distance observations derived from the compass to the acoustic network adjustment and combined the observations from both the forward and rear networks for adjustment calculation to improve positioning accuracy in 2013 [20]. Although multi-streamer numerical positioning methods are widely used, they still have some limitations. First, such methods do not establish a direct relationship between the positioning data and hydrophone coordinate positions, and the modeling and calculation processes are relatively imprecise. During data processing, multi-source data are often used separately, and effective fusion processing is not achieved. Second, segmenting the towed streamer using circular arcs or piecewise lines may cause discontinuities at connection points. This does not reflect the actual smooth curvature of the streamer, thereby reducing positioning and model accuracy. Currently, towed streamer exploration is gradually shifting toward four-dimensional dynamic monitoring scenarios. This shift imposes higher requirements on towed streamer positioning accuracy, stability, and processing efficiency. Under this trend, multi-streamer analytical positioning is gradually becoming the mainstream method for towed streamer positioning. Multi-streamer analytical positioning constructs a mathematical model of the towed streamer, closely links various parameters to multiple observations, and jointly solves for streamer shape parameters to determine hydrophone coordinates. For example, Yu et al. proposed a multi-streamer analytical positioning method based on polynomial curve fitting in 2022, establishing a polynomial curve model for the three-dimensional coordinate components of each towed streamer. However, under conditions of large curvature turns, this model sometimes exhibits significant modeling errors [21,22]. Duan et al. proposed a rigorous curve integration model in 2022, which establishes a rigorous integration relationship between each point of the towed streamer based on compass observations. Nevertheless, due to the integration operation involving unknown original functions, the solution efficiency is slightly low [23,24,25]. Sang et al. introduced wave motion harmonic components and proposed a hybrid harmonic function positioning model in 2024, which effectively reduces the modeling errors of the polynomial curve model during turns, but some modeling errors still exist during high-curvature turns [26]. In addition, the processing of observations such as sound velocity and magnetic declination also affects positioning accuracy, and these two types of positioning observations are mainly affected by the marine environment [27,28]. It is still necessary to refine the processing of observations such as sound velocity and magnetic declination in order to obtain more stable results. Given the above shortcomings of existing geometric positioning models, this paper proposes a towed streamer analytical positioning model based on spline curve fitting. The model performs segmented streamer fitting and derives observation error equations using cubic polynomials as an example to enhance positioning accuracy and reliability. The superiority of the spline curve model is verified through simulations and field data.

## 2. Spline Curve Fitting Method

**Figure 1:**
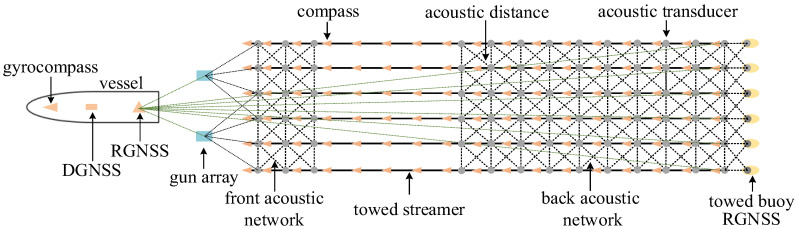
Offshore seismic exploration towed streamer positioning network.

In marine towed streamer seismic exploration, the towed streamer positioning network comprises positioning equipment such as a DGNSS (Differential Global Navigation Satellite System), RGNSS (Relative GNSS), gyrocompass, acoustic transducer, and compass, as shown in Figure 1. The DGNSS provides an absolute position reference for the entire positioning network. After the vessel’s heading is determined by the gyrocompass, the coordinates of any node on the vessel can be calculated. The RGNSS transmits the position reference to the gun array and tail buoy, providing a reference point for the underwater towed streamer positioning. Acoustic observations form an acoustic observation network. Combined with compass and depth observations, this determines the shape and position of the towed streamer [29,30]. Each streamer is equipped with hydrophones spaced approximately 10 m apart to collect seismic wave data. Determining the positioning information of these hydrophone arrays is equivalent to determining the shape of the entire streamer. Thus, the streamer positioning problem is essentially a streamer shape determination.

### 2.1. Spline Curve Model

Given the smooth geometric properties of the underwater towed streamer, polynomial curve fitting models are suitable for streamer positioning. Since the streamer depth is controlled by the compass and is relatively stable, polynomial curve models are generally established for the directional coordinate components of each streamer. Observations from compasses and acoustic transducers are utilized for parameter estimation. However, while a single polynomial curve can describe the shape of the towed streamer, the modeling error is relatively large under high-curvature turning conditions. Additionally, high-order polynomials are prone to the “Runge phenomenon,” where high-order term oscillations cause the fitted curve to deviate from the actual streamer shape. The spline curve model performs segmented fitting of the curve. According to this model, the coordinates at any point along the towed streamer can be expressed as follows:

(1) $$\left\{\begin{array}{c} n(s)=f_{n}(s)=\sum_{k=0}^{n}a_{k}s^{k}=S^{T}A\\ e(s)=f_{e}(s)=\sum_{k=0}^{n}b_{k}s^{k}=S^{T}B \end{array}\right.$$

where $S={\left[s^{0},s^{1},\cdots ,s^{n}\right]}^{T}$, n is the polynomial order, and $A={\left[a_{0},a_{1},\cdots ,a_{n}\right]}^{T}$ and $B={\left[b_{0},b_{1},\cdots ,b_{n}\right]}^{T}$ are the coefficients of the polynomial.

Since each segment of the towed streamer is fitted using a spline curve, ensuring a smooth transition at the connection points between segments is essential. Thus, continuity constraints are added at the boundaries of each spline curve segment. Specifically, the adjacent spline segments have continuous and numerically equal zero-order, first-order, and second-order derivatives, ensuring continuity and smoothness at the connection points [31]. The constraint conditions in each coordinate component direction can be expressed as following:

(2) $$\left\{\begin{array}{l} f_{i}(s_{i,i+1})=f_{i+1}(s_{i,i+1})\\ f_{i}^{\prime }(s_{i,i+1})=f_{i+1}^{\prime }(s_{i,i+1})\\ f_{i}^{\prime \prime }(s_{i,i+1})=f_{i+1}^{\prime \prime }(s_{i,i+1}) \end{array}\right.$$

Among them, $s_{i,i+1}$ is the mileage value at the connection between the i-th and (i+1)-th segment of the towed streamer, and $f_{i}$ and $f_{i+1}$ are the spline curves of the i-th and the (i+1)-th segment of the streamer, respectively, representing the zero-order continuity condition. $f_{i}^{\prime }(s_{i,i+1})$ and $f_{i+1}^{\prime }(s_{i,i+1})$ are the first-order derivatives of the spline curves of the i-th and the (i+1)-th segment, respectively, representing the first-order continuity condition. $f_{i}^{\prime \prime }(s_{i,i+1})$ and $f_{i+1}^{\prime \prime }(s_{i,i+1})$ are the second-order derivatives of the spline curves of the i-th and the (i+1)-th segment, respectively, representing the second-order continuity condition.

### 2.2. Observation Error Equations

The most typical spline curve is the cubic spline curve, which combines multiple cubic polynomial segments into a composite curve, effectively describing complex curve shapes. In this study, cubic polynomials are employed as a representative case to model towed streamers. To solve for the coefficients of the segmented cubic spline curve model established above, it is necessary to establish the mathematical relationship between the observations (e.g., coordinates, compass azimuth, and acoustic transducer distance) and the cubic spline curve coefficients. The error equations for various types of observations are derived as follows:

**Coordinate observation error equation**: Including the coordinates of the front and rear buoys (i.e., head buoy and tail buoy) of the streamer, as well as the virtual coordinates predicted based on the status information of the compass and acoustic nodes on the streamer, the coordinate equation of the i-th node on the towed streamer is as follows:

(3) $$x_{i}=f(s_{i})={\left[\begin{array}{cc} S_{i} & \\ & S_{i} \end{array}\right]}^{T}\left[\begin{array}{c} A\\ B \end{array}\right]$$

In the formula, $x_{i}$ is the coordinate vector of node i, $s_{i}$ is the designed mileage value of node i on the towed streamer, and the meanings of the other parameters are the same as in Formula (1).

Based on the approximate coordinates of each node on the streamer, the coordinate components in the n and e directions can be obtained, along with the approximate values of the corresponding parameters $A^{0}$ and $B^{0}$ to be estimated. Based on the approximate values of the parameters, Equation (3) can be linearized:

(4) $$\left[\begin{array}{c} v_{n_{i}}\\ v_{e_{i}} \end{array}\right]={\left[\begin{array}{cc} S_{i} & \\ & S_{i} \end{array}\right]}^{T}\left[\begin{array}{c} \delta A\\ \delta B \end{array}\right]-\left[\begin{array}{c} l_{n_{i}}\\ l_{e_{i}} \end{array}\right]$$

In the equation, δA and δB are the correction values of the estimated parameters, and $l_{n_{i}}$ and $l_{e_{i}}$ are constant terms.

**Azimuth observation error equation**: Compasses, which are installed at intervals of several hundred meters along the streamer, are primarily used to measure the magnetic azimuth of the tangent direction at each node. The compass observations at the towed streamer after magnetic declination correction can be expressed as follows:

(5) $$\alpha_{s_{i}}=\arctan\left(\frac{f_{e}^{\prime }(s_{i})}{f_{n}^{\prime }(s_{i})}\right)=\arctan\frac{D^{T}B}{D^{T}A}$$

In the formula, $f_{n}^{\prime }(s_{i})$ and $f_{e}^{\prime }(s_{i})$ are the derivatives of the polynomial functions of the n and e coordinate components at the towed streamer, $D={\left[0,s,2s,\cdots ,ns^{n-1}\right]}^{T}$, and then the arctangent function is linearized according to the Taylor series expansion:

(6) $$\begin{array}{c} v_{\alpha_{s_{i}}}=\frac{-D^{T}B^{\circ }D^{T}}{D^{T}\left(A^{\circ }{\left(A^{\circ }\right)}^{T}+B^{\circ }{\left(B^{\circ }\right)}^{T}\right)D}\delta A\\ \;\;\;\;+\frac{D^{T}A^{\circ }D^{T}}{D^{T}\left(A^{\circ }{\left(A^{\circ }\right)}^{T}+B^{\circ }{\left(B^{\circ }\right)}^{T}\right)D}\delta B-l_{\alpha_{s_{i}}} \end{array}$$

In the formula, $v_{\alpha_{s_{i}}}$ is the correction value for the compass observations at point $s_{i}$ on the towed streamer, and $l_{\alpha_{s_{i}}}=\alpha_{s_{i}}-\alpha_{s_{i}}^{\circ }$ is the constant term. $\alpha_{s_{i}}^{\circ }$ is the bearing calculated from the approximate value of the towed streamer coefficient, and the meanings of the other parameters are the same as in Formulas (4) and (5).

**Acoustic distance error observation equation**: The acoustic distance is obtained from two acoustic transducers on the streamer, including two types of acoustic distances: same streamer and different streamer. For generality, assume that $r_{j,k}$ is the acoustic distance between the j-th acoustic transducer on the g-th streamer and the k-th acoustic transducer on the h-th streamer. Its observation equation is as follows:

(7) $$r_{j,k}={\left[{\left(S_{k}^{T}A_{h}-S_{j}^{T}A_{g}\right)}^{2}+{\left(S_{k}^{T}B_{h}-S_{j}^{T}B_{g}\right)}^{2}\right]}^{\frac{1}{2}}$$

In the formula, $S_{k}={\left[s_{k}^{0},s_{k}^{1},\cdots ,s_{k}^{n}\right]}^{T}$, $S_{j}={\left[s_{j}^{0},s_{j}^{1},\cdots ,s_{j}^{n}\right]}^{T}$, $A_{g}$ and $B_{g}$ are the polynomial coefficients of the towed streamer g, and $A_{h}$ and $B_{h}$ are the polynomial coefficients of the towed streamer h. Linearizing the above formula yields the following:

(8) $$\begin{array}{c} v_{r_{j,k}}=\frac{(S_{j}^{T}A_{g}^{\circ }-S_{k}^{T}A_{h}^{\circ })S_{j}^{T}}{r_{i,j}^{\circ }}\delta A_{g}+\frac{(S_{k}^{T}A_{h}^{\circ }-S_{j}^{T}A_{g}^{\circ })S_{k}^{T}}{r_{i,j}^{\circ }}\delta A_{h}\\ \;\;\;\;+\frac{(S_{j}^{T}B_{g}^{\circ }-S_{k}^{T}B_{h}^{\circ })S_{j}^{T}}{r_{j,k}^{\circ }}\delta B_{g}+\frac{(S_{k}^{T}B_{h}^{\circ }-S_{j}^{T}B_{g}^{\circ })S_{k}^{T}}{r_{j,k}^{\circ }}\delta B_{h}-l_{r_{j,k}} \end{array}$$

In the formula, $v_{r_{j,k}}$ is the acoustic distance observation correction value, and $A_{g}^{\circ },B_{g}^{\circ }$ and $A_{h}^{\circ },B_{h}^{\circ }$ are the polynomial coefficient approximations of towed streamer g and towed streamer h, respectively. $\delta A_{g},\delta B_{g}$ and $\delta A_{h},\delta B_{h}$ are the corresponding polynomial coefficient correction values, $r_{j,k}^{\circ }$ is the acoustic distance calculated based on the towed streamer coefficient approximation, and $l_{r_{j,k}}=r_{j,k}-r_{j,k}^{\circ }$ is the constant term.

**Boundary continuity constraint equation**: To ensure smooth continuity at the connection points of the spline curve, the boundary continuity constraint conditions need to be listed as conditional equations and included in the least squares adjustment. The constraint equations for the n and e direction coordinate components can be expressed as follows:

(9) $$\left\{\begin{array}{l} S_{i,i+1}^{T}A_{i+1}-S_{i,i+1}^{T}A_{i}=0\\ E_{i,i+1}^{T}A_{i+1}-E_{i,i+1}^{T}A_{i}=0\\ F_{i,i+1}^{T}A_{i+1}-F_{i,i+1}^{T}A_{i}=0 \end{array}\right.$$

(10) $$\left\{\begin{array}{l} S_{i,i+1}^{T}B_{i+1}-S_{i,i+1}^{T}B_{i}=0\\ E_{i,i+1}^{T}B_{i+1}-E_{i,i+1}^{T}B_{i}=0\\ F_{i,i+1}^{T}B_{i+1}-F_{i,i+1}^{T}B_{i}=0 \end{array}\right.$$

Among them, $A_{i}$ and $B_{i}$ are the cubic polynomial coefficients in the n and e directions of the i-th segment of the streamer, while $A_{i+1}$ and $B_{i+1}$ correspond to the (i+1)-th segment, respectively, with $S_{i,i+1}={\left[1,s_{i,i+1},s_{i,i+1}^{2},s_{i,i+1}^{3}\right]}^{T}$, $E_{i,i+1}={\left[0,1,2s_{i,i+1},3s_{i,i+1}^{2}\right]}^{T}$, $F_{i,i+1}={\left[0,0,2,6s_{i,i+1}\right]}^{T}$. The above constraint equations are linear and do not need to be linearized.

### 2.3. Hydrophone Position Estimation

The hydrophone position calculation process includes pre-processing and adjustment calculations. First, the towed streamer positioning data are pre-processed, primarily for observation quality control and data epoch synchronization [32]. Then, spline interpolation is used to estimate the azimuth of any point. Using the approximate coordinates of the acoustic transducer node and compass node obtained from coordinate integration, polynomial fitting is used to derive the initial values of the streamer curve coefficients. Finally, the least squares adjustment is used to estimate the correction values of the parameters:

(11) $$\delta \hat{X}={\left(H^{T}PH\right)}^{-1}H^{T}PL$$

In the formula, $\delta \hat{X}$ is the correction value of the spline curve coefficient to be estimated; H, L, and P are the design matrix, observation vector, and weight matrix, respectively, obtained from the above observation error equation.

Considering that spline models require more parameters to be estimated than traditional polynomial vectors, it is generally necessary to use approximate coordinates of nodes such as compass nodes as virtual observations in the adjustment. This avoids rank deficiency in the normal equations and improves the stability of solving the curve coefficients. The stochastic model can be determined based on the nominal accuracy of the sensor. For example, the prior accuracy of RGNSS coordinate observations is generally set to 0.5–1 m, the prior accuracy of acoustic distance observations is approximately 2–3 m, and the prior accuracy of compass bearing observations is approximately 1°. The weights can also be determined based on variance component estimation [22].

Taking into account the constraint Equations (9) and (10), they are converted to

(12) $$D\delta \hat{X}-L_{X}=0$$

In the formula, D represents the coefficient of the constraint equation of the boundary condition, and $L_{X}$ represents the observed minus calculated value of the constraint equation.

A joint least squares adjustment incorporating the constraint equations is performed, and the equation is solved using an additional-constraint approach. When correcting spline curves, this is expressed as follows:

(13) $$\delta \hat{X}=\left(N^{-1}-N^{-1}D^{T}N_{DD}^{-1}DN^{-1}\right)H^{T}PL+N^{-1}D^{T}N_{DD}^{-1}L_{X}$$

In the formula, $N=H^{T}PH$, $N_{DD}=DN^{-1}D^{T}$, D is the coefficient matrix of the constraint equation. The updated spline coefficient $\delta \hat{X}$ of the towed streamer is calculated, and this updated $\hat{X}$ is used as the approximate value $X^{\circ }$ for the new iteration. P is the weight matrix. The above process is repeated until the correction value $\delta \hat{X}$ is less than a given threshold.

After determining the spline coefficients of the towed streamer, the mileage value of any node is substituted into its corresponding segmented polynomial to obtain the coordinates of that node. The variance-covariance matrix $D_{\left(n,e\right)}$ of the coordinates (n,e) of each point calculated by Formula (1) can be obtained by the following formula:

(14) $$D_{\left(n,e\right)}={\hat{\sigma }}_{0}^{2}\Phi Q_{\hat{X}\hat{X}}\Phi^{T}$$

In the formula, ${\hat{\sigma }}_{0}^{2}$ is the a posteriori variance factor; Φ is the design mileage matrix corresponding to coordinate, i.e., the coefficient matrix in Formula (1); and $Q_{\hat{X}\hat{X}}={\left(H^{T}PH\right)}^{-1}$ is the co-factor matrix of parameter $\hat{X}$.

The parameters of the model are also key to the efficiency of the solution. The number of parameters for polynomial curve model and cubic spline model are $n_{dim}\times n_{ts}\times (n_{ord}+1)$ and $n_{dim}\times n_{ts}\times n_{seg}\times 4$, respectively, where $n_{dim}$ is the dimension, $n_{ts}$ is the number of towed streamers, $n_{ord}$ is the order, and $n_{seg}$ is the number of segments. The parameters of the cubic spline model are slightly higher than those of the polynomial curve model, and the polynomial curve model is slightly more efficient in solving, but both models can meet the actual exploration needs.

## 3. Experiments and Results Analysis

To evaluate the positioning accuracy and stability of the spline model proposed in this paper, simulation and field measurement data were used for verification, and the results were compared and analyzed with those of traditional polynomial curve models.

### 3.1. Simulation Experiment

**Figure 2:**
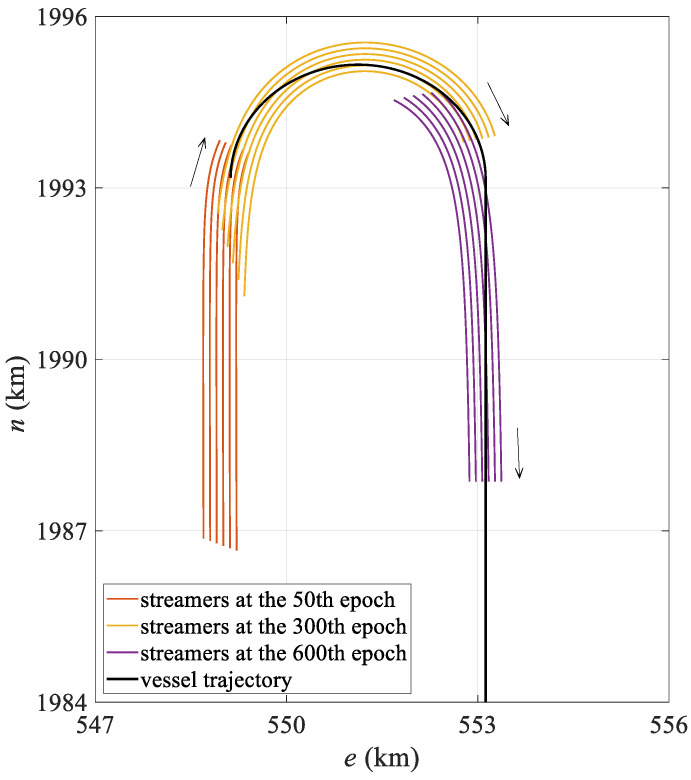
Simulated vessel trajectory and streamer shape at different epochs.

The simulation data were generated using SimStr 1.0, a towed streamer simulation platform developed by the author’s team [33]. Based on a towed streamer dynamics model, this platform accurately simulates the underwater motion of streamers and outputs P2/94 and P1/90 files, which are standard data exchange formats used in marine seismic exploration and are used to evaluate towed streamer positioning models. The simulated towed streamer positioning network comprises six streamers spaced 100 m apart, each with a length of 7 km. Each towed streamer is equipped with 561 hydrophones spaced 12.5 m apart and 25 compasses spaced 300 m apart, plus an additional compass attached to the tail of the streamer. The commonly used front–rear acoustic network configuration was adopted. Two acoustic transducers spaced 75 m apart were mounted at the head of each streamer to form the front acoustic network with the gun array acoustic transducers. Starting from 3500 m along the towed streamer mileage, 18 acoustic transducers spaced 200 m apart were mounted at the rear of each streamer to form the rear acoustic network with the tail acoustic transducers. In addition, DGNSS and gyrocompasses were installed on the survey vessel, and an RGNSS was installed on both the gun array and the tail buoy. At a vessel speed of 2 m/s, the towed streamer continued to move for 10,000 s, of which the first 7000 s were used to complete a 180° turn with a turning radius of 2000 m, and the last 3000 s were in a straight line. The simulation platform recorded observations at 10 s intervals, storing data to the P2/94 file while recording the actual coordinates of the hydrophone to the P1/90 file, recording a total of 1000 observation epochs. Figure 2 shows the streamer shape during the turning state of the first 700 epochs.

Based on simulation data, the two models in this paper were tested. The polynomial curve model was set to seventh order, and in the spline curve model, the streamer was divided into seven ~1 km segments based on mileage, each represented by a cubic spline. The coordinates of the hydrophone at each epoch obtained by solving the two positioning models were compared with the actual hydrophone coordinates in the P1/90 file output by SimStr, and the mean position deviation of the hydrophone at each epoch was calculated as follows:

(15) $$d^{i}=\frac{1}{n_{r}}\sum_{j=1}^{n_{r}}{\left[{\left({\hat{n}}_{j}^{i}-{\tilde{n}}_{j}^{i}\right)}^{2}+{\left({\hat{e}}_{j}^{i}-{\tilde{e}}_{j}^{i}\right)}^{2}\right]}^{\frac{1}{2}}$$

Here, $d^{i}$ is the mean positioning deviation of the i-th epoch, $\left({\hat{n}}_{j}^{i},{\hat{e}}_{j}^{i}\right)$ is the positioning result of hydrophone in the i-th epoch, $\left({\tilde{n}}_{j}^{i},{\tilde{e}}_{j}^{i}\right)$ is the true coordinate of hydrophone in the i-th epoch recorded in the simulated P1/90 file, $n_{r}=n_{str}\times n_{hydrophone}$ is the total number of hydrophones, $n_{str}$ is the number of towed streamers, and $n_{hydrophone}$ is the number of hydrophones on each towed streamer.

During real-time towed streamer calculation, it is usually required that the calculation be completed within the time between two adjacent shots, which takes about ten seconds. In fact, it is easy to meet this operational requirement for each shot, so this paper will not discuss calculation efficiency.

**Figure 3:**
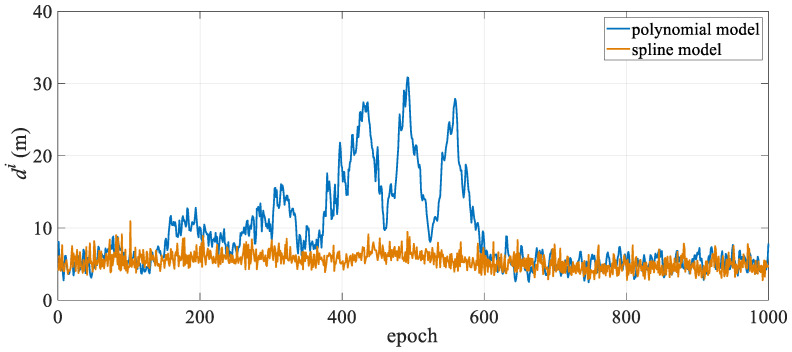
Mean hydrophone position deviation over time.

The mean positioning deviation of the hydrophone for each epoch was calculated and statistically analyzed, with the results presented in Table 1. The table shows that in the turning state, the polynomial curve model has a mean hydrophone deviation of 10.86 m and a maximum deviation exceeding 30 m, while the spline curve model has a mean hydrophone deviation of only 5.74 m, representing a 47.1% reduction in modeling error compared to the polynomial curve model. Moreover, in the turning state, the spline model exhibits significantly lower maximum and standard deviations than the polynomial curve model, indicating more stable positioning results. From the mean positioning deviation diagram for all hydrophones in each epoch shown in Figure 3, it is evident that the polynomial curve model exhibits significant fluctuations between epochs 150 and 600. As shown in Figure 2, the towed streamer was in a turning state for the first 700 epochs, with smaller curvatures in the first 50 epochs and after the 600th epoch, and transitioned to a straight state after approximately the 700th epoch. The periods of largest positioning deviation correspond to the time periods with the most significant changes in the curvature of the streamer. The polynomial curve model shows significant deviations during turning, primarily due to modeling errors. The spline curve model maintains stable positioning deviation throughout the entire time sequence with virtually no fluctuations, and the results are significantly better than those of the polynomial curve model. This demonstrates that when the towed streamer has a large curvature, the spline model better adapts to dynamic changes in the streamer’s shape, more effectively reduces modeling errors, and enhances the positioning accuracy and stability of the towed streamer. Additionally, in the straight-line state, the positioning results of the two models are essentially consistent and can be regarded as equivalent.

To quantitatively analyze the deviation changes across different towed streamer mileage sections and further compare the spatial positioning accuracy of the two models, Formula (16) was used to calculate the towed streamer positioning deviation $d_{j}$ at different mileage sections.

(16) $$d_{j}=\frac{1}{n_{t}}\sum_{i=1}^{n_{t}}{\left[{\left({\hat{n}}_{j}^{i}-{\tilde{n}}_{j}^{i}\right)}^{2}+{\left({\hat{e}}_{j}^{i}-{\tilde{e}}_{j}^{i}\right)}^{2}\right]}^{\frac{1}{2}}$$

In the formula, $n_{t}$ is the total number of observation epochs, and $d_{j}$ is the mean hydrophone deviation at the same mileage for all towed streamers.

**Figure 4:**
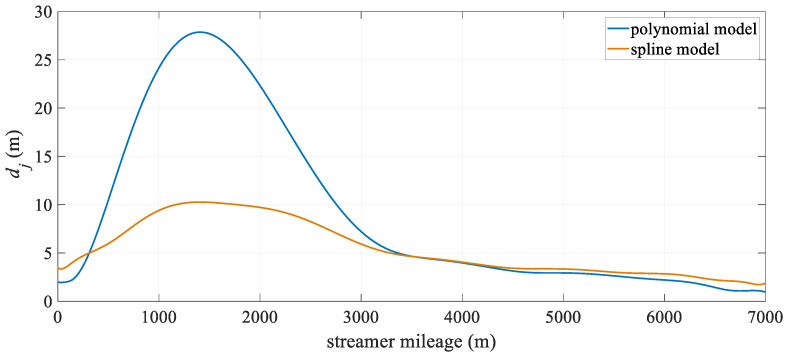
Mean positioning deviation of hydrophone at different streamer mileage.

Figure 4 illustrates the change in hydrophone deviation with towed streamer mileage. It is evident that both models exhibit relatively large positioning deviations in the middle section of the towed streamer. This is because the front section of the streamer only has compass observations and lacks acoustic observations. Additionally, the front and rear sections of the streamer have high-precision RGNSS coordinate observations, which results in the front section of the streamer having significantly lower positioning accuracy than the two ends. Overall, the spline model outperforms the polynomial curve model, particularly in the middle-front section of the streamer, where there are no acoustic observations. After 4000 m, the positioning accuracy of the polynomial curve model approaches that of the spline model. This suggests that the spline model can significantly reduce modeling errors in the absence of acoustic observations, while at other mileage sections, its positioning accuracy remains essentially the same as that of the polynomial curve model.

**Figure 5:**
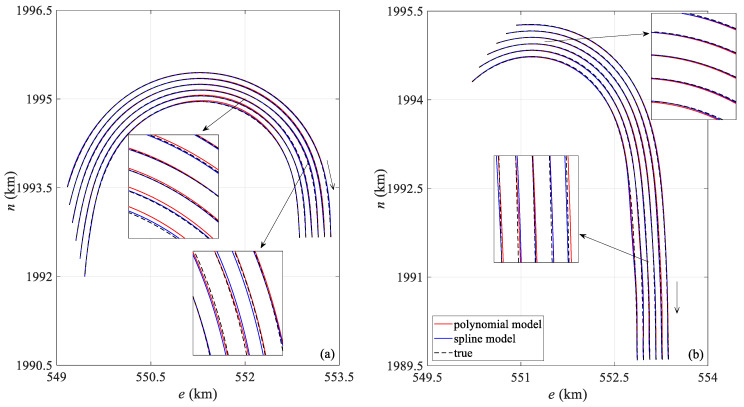
Comparison of streamer shapes by two positioning models: (**a**) the 360th epoch, (**b**) the 513th epoch.

Figure 5 shows a comparison between the streamer shape calculated by the two positioning models at the 360th and 513th epochs during the towed streamer turn and the actual streamer shape. In Figure 5a, the curvature of all sections of the towed streamer is approximately uniform, while in Figure 5b, the curvature of the front section of the towed streamer is small, and the sections with larger curvatures are mainly concentrated in the rear. In Figure 5a, the polynomial curve model shows significant differences from the actual streamer shape in the front and middle sections, whereas the spline model closely matches the actual streamer shape. In Figure 5b, both models show some deviation from the actual streamer shape, but the spline model is closer to the actual shape, while the polynomial curve model exhibits the largest deviation in the front and middle sections. The main reason is that the front and middle sections lack acoustic network constraints, leading to error accumulation, a phenomenon also observed in Figure 4. Regarding the shape of each individual streamer, the shapes of the first four streamers calculated by the two models (numbered from the starboard side of the seismic survey vessel; this applies to other streamer shape comparison figures in this paper as well) are consistent with the actual streamer shapes, but the shapes of the last two towed streamers calculated by the polynomial curve model have significant positioning deviations from the actual streamer shapes. The main reason is that the curvature of the last two towed streamers is significantly greater than that of the first four. The polynomial curve model has a large modeling error in large curvature turns, leading to obvious distortion of the streamer shape.

### 3.2. Field Experiment

In order to verify the practical application effect of the spline curve positioning model proposed in this paper, tests were conducted using field data from two operational scenarios, one with six streamers and one with ten streamers. As the actual hydrophone coordinates are not available in the field measurement data, this paper compares the results of the two models with results from international commercial towed streamer positioning software.

#### 3.2.1. Case I: 6-Streamer Scenario

**Figure 6:**
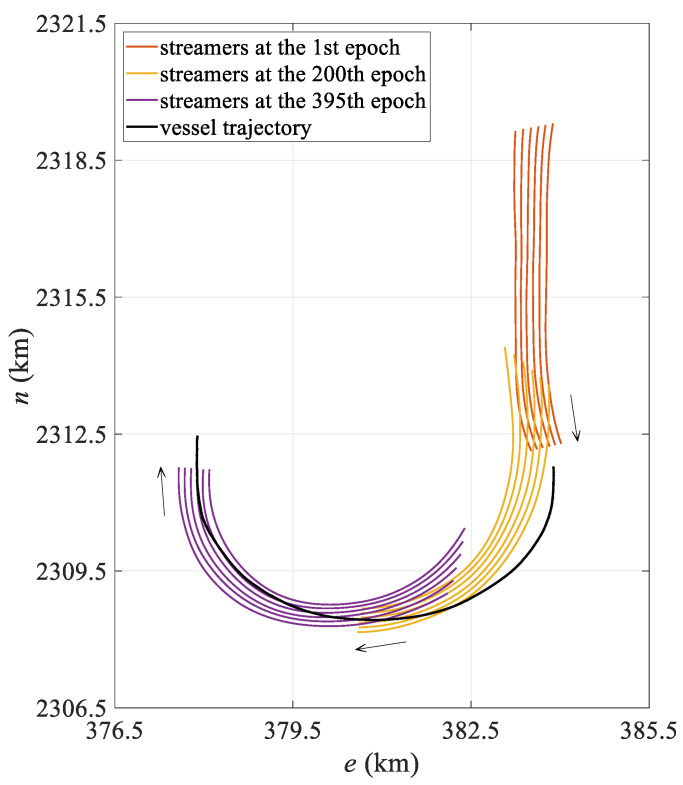
Case I: Vessel trajectory and streamer shape at different epochs.

The field measurement data were acquired from an actual offshore seismic exploration operation conducted in October 2024. The towed streamer vessel operation configuration is the same as the simulated six-streamer data, except that each streamer is equipped with 33 compasses, and the towed streamer turning radius is approximately 2500 m. Figure 6 shows the movement trajectory of the vessel and the position of the towed streamer at different epochs in the measured data. The towed streamer turning data lasted for about 4000 s, during which the towed streamer vessel and the head of the towed streamer completed a 180° turn, while the rear of the towed streamer completed a 90° turn.

The above data were processed using two positioning models and international commercial towed streamer positioning software. Equations (15) and (16) were used to calculate the hydrophone positioning deviations of the two positioning models from the reference value over time. Table 2 presents the statistical results of the positioning deviations between the two positioning models and the reference values. The results show that the mean error of the polynomial curve model is 16.74 m, while that of the spline model is 13.39 m, representing an improvement in accuracy of approximately 20.0%. The maximum temporal deviation decreased from 33.81 m to 28.65 m, and the standard deviation of the spline model was smaller than that of the polynomial curve model, indicating that the spline curve model has superior positioning accuracy and stability compared to the polynomial curve model.

**Figure 7:**
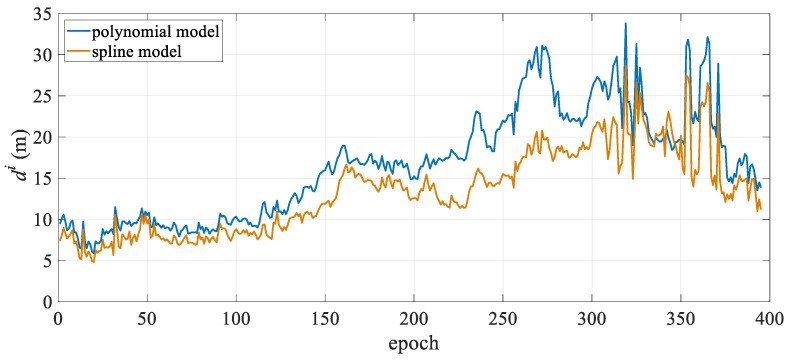
Case I: Mean positioning deviation of hydrophones per epoch.

Figure 7 shows the mean positioning deviation of the hydrophone for each observation epoch. It can be seen that the deviation of the two positioning models for the first 200 epochs is approximately uniform, with minimal deviation from the reference value. The positioning deviation of the two models for the last 200 epochs is greater than that for the first 200 epochs, and the positioning accuracy of the spline curve is significantly better than that of the polynomial curve model. After 300 epochs, both models exhibit jumps to different degrees, likely due to variations in observation quality. Both models apply single-epoch calculations and lack temporal continuity constraints.

**Figure 8:**
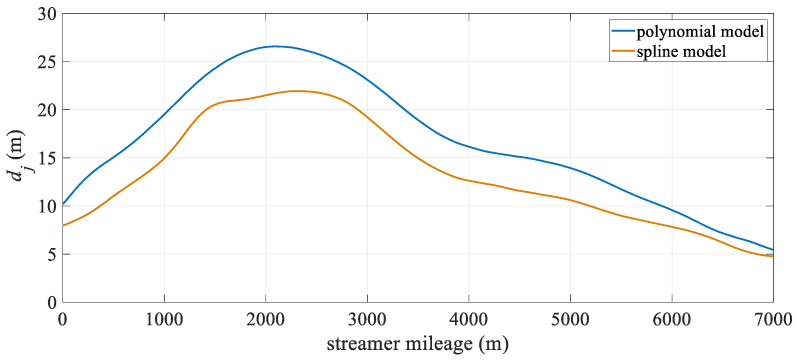
Case I: Mean positioning deviation of hydrophones at different mileages.

**Figure 9:**
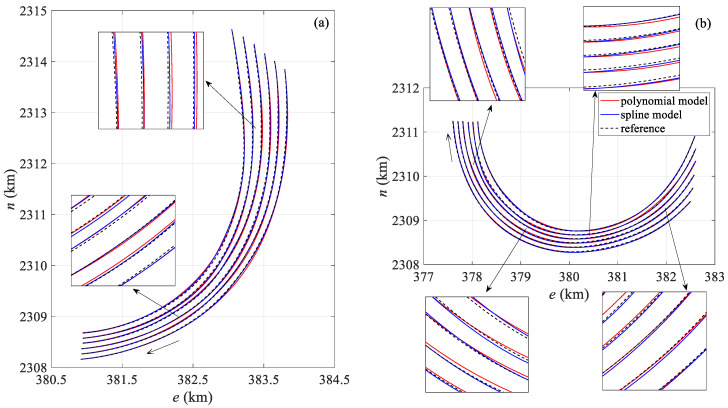
Case I: Comparison of streamer shapes by two positioning models: (**a**) the 190th epoch, (**b**) the 372nd epoch.

Figure 8 shows the deviation of the two models at different distances along the towed streamer. The spline model exhibits significantly smaller deviations than the polynomial curve model, reducing the modeling error, particularly in the front and middle sections. As shown in Table 2, the maximum spatial deviation decreased from 26.56 m to 21.94 m. Combined with the large curvature turn in the latter half of Figure 9, it is evident that in scenarios with large turn curvature, the spline curve model achieves a more significant improvement in positioning accuracy. Both positioning models exhibited the largest positioning deviations in the front section of the towed streamer, where there were no acoustic observations. Similar to the reasons mentioned above, this was related to the front-rear net configuration.

Figure 9 compares the streamer shapes at the 190th and 372nd epochs. Compared with the polynomial curve model, the spline model is closer to the reference streamer shape in both epochs. In Figure 9a, the polynomial curve model exhibits greater deviation than the spline model in the middle and rear sections of the streamer. On the one hand, the middle section is constrained only by the compass. On the other hand, the noise in the compass and acoustic observations in the rear of the towed streamer are significantly greater in this set of data, and there are significant missing values in the acoustic observations, resulting in different degrees of deviation in both models.

#### 3.2.2. Case II: 10-Streamer Scenario

**Figure 10:**
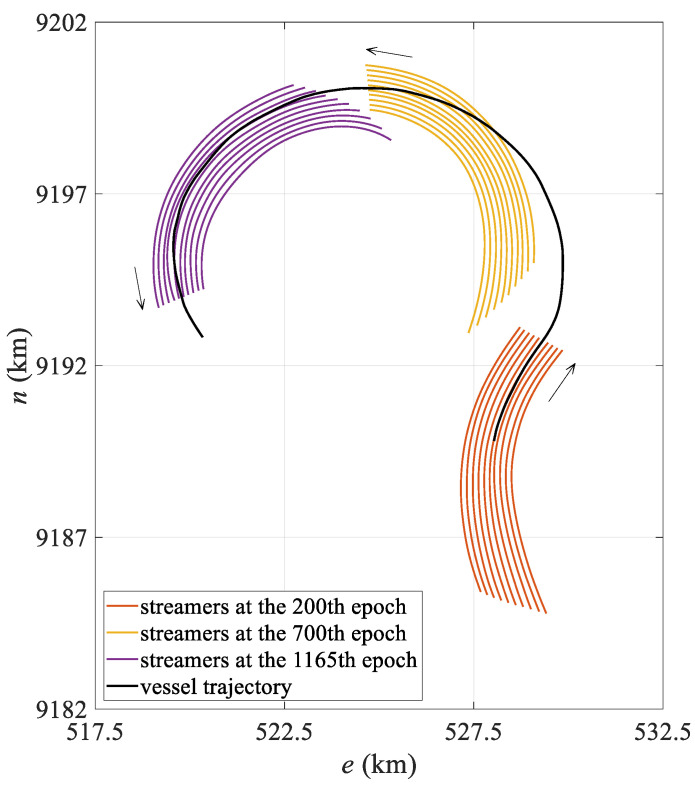
Case II: Vessel trajectory and streamer shape at different epochs.

The field measurement data originate from an offshore exploration operation conducted in 2024. According to the actual exploration operation requirements, the vessel is equipped with three DGNSS devices, two gyrocompasses, and one RGNSS reference station to provide position calculation references for the towed streamer positioning network. Meanwhile, the vessel tows 10 streamers, each 8100 m in length, with a spacing of 150 m between adjacent streamers. Each towed streamer is equipped with 648 hydrophones, 39 compasses, 19 acoustic transducers. The hydrophones are spaced 12.5 m apart. The first three compasses are spaced at 75 m and 150 m intervals. The remaining compasses are spaced 300 m apart. The acoustic transducers are configured in a front–rear network configuration. Three acoustic transducers are deployed at the front of the towed streamer, forming a front network acoustic array with the gun array and the acoustic transducer on the head buoy. Sixteen acoustic transducers in the middle and rear sections of the towed streamer, along with the acoustic transducer on the tail buoy, form a rear network acoustic array. As shown in Figure 10, the measured data records the entire process of the towed streamer turning 180°. The turning radius was approximately 3500 m, and the entire turn lasted approximately 3.3 h and comprised a total of 1165 epochs.

Table 3 shows the mean, standard deviation, maximum, and minimum values of the mean positioning deviation of the two models by epoch. The results indicate that the point error of the spline curve model is 35.0% lower than that of the polynomial curve model, with the maximum value decreasing from 37.30 m to 28.92 m. This demonstrates that the spline curve model provides superior positioning accuracy and stability compared to the polynomial curve model.

**Figure 11:**
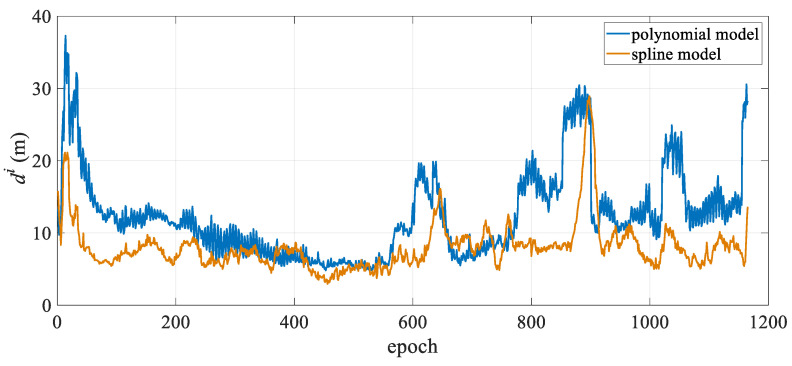
Case II: Mean positioning deviation of hydrophones per epoch.

Figure 11 shows the mean positioning deviation of the hydrophone in each epoch. Both models show peaks in the first 100 epochs and the last 600 epochs. Combined with the streamer trajectory diagram in Figure 10, the main reason for the peaks in the first 100 epochs is likely the significant curvature changes in the towed streamer, particularly in the middle and rear sections, as it turned from east to west. This resulted in a substantial increase in modeling errors. In the last 600 epochs, the curvature of the towed streamer’s turn increased slightly, and the quality of the acoustic observations during this period became poor, particularly in the middle and rear sections, leading to multiple peaks. Overall, the polynomial curve model has certain fluctuations, while the deviation of the spline model is relatively stable.

**Figure 12:**
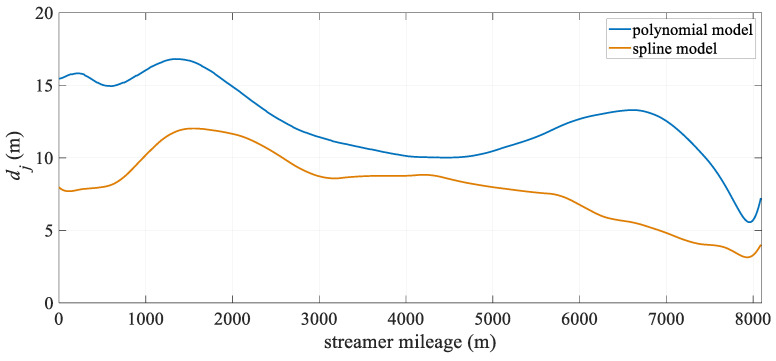
Case II: Mean positioning deviation of hydrophones at different streamer mileages.

Figure 12 shows the positioning deviation of the two models at different distances. It can be seen from the figure that the deviation of the polynomial curve model is mainly concentrated in the front and rear sections of the towed streamer, while the deviation of the spline model is mainly concentrated in the front section. This is primarily due to the front-rear network configuration but may also result from differences in the processing of acoustic observations and commercial hydrophone positioning data. Unlike the polynomial curve model, which exhibits no obvious patterns in its deviation changes, the spline model shows smaller deviations in the middle-rear section and a significant reduction in the front-middle section. Overall, the deviation distribution is more uniform, indicating higher spatial stability in the positioning results.

**Figure 13:**
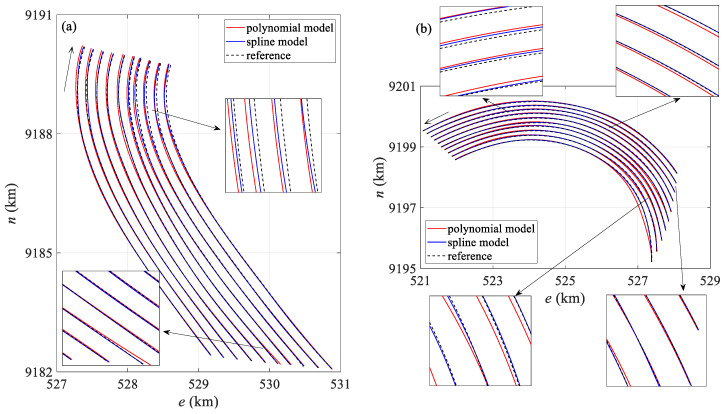
Case II: Comparison of streamer shapes by two positioning models: (**a**) the 50th epoch, (**b**) the 866th epoch.

Figure 13 presents the comparison results between the two models and the reference streamer shape at the 50th and 866th epochs during the towed streamer turn. Figure 13a shows that the inner streamer shapes of both models differ from the true values. Compared with the spline curve model, the polynomial curve model shows greater discrepancies from the reference streamer shape, primarily in the front and middle segments. The main reason is that the curvature of the rear section of the streamer in this epoch is significantly smaller than that of the front section, which may be largely attributed to modeling errors. Figure 13b shows the comparison of streamer shapes at epoch 866. The curvature of each section of the towed streamer in this epoch is similar. When the turning curvature is large, the comparison results of the two models with the reference value show that the spline curve model is largely consistent with the reference streamer shape, while the polynomial curve model exhibits certain deviations from the reference value. The difference between the inner and outer towed streamers is greater on the inner side, mainly due to the greater curvature of the inner towed streamer when turning. In addition, there is a large deviation at the tail of the innermost 10th streamer, and the streamer shape calculated by the polynomial curve model deviates significantly from the reference streamer shape. This may be due to the significant number of missing acoustic values in this section. The polynomial curve model involves a global adjustment of the entire network and lacks the segmented constraints of the spline model, resulting in this section not achieving local optimization.

## 4. Discussion

Reliable seismic data, efficient survey line changes, and reduced operational risk all depend on the positioning accuracy and stability of the towed streamer in marine seismic exploration. Currently, polynomial curve models are commonly used for towing streamer modeling, which can effectively fit the shape of streams during towing operations, but significant modeling errors may occur when streamers turn, especially in scenarios involving high-curvature turns. The arc segment model, curvilinear integral model, and hybrid harmonic curve model can effectively reduce modeling errors, but each has its own limitations. Numerical methods based on arc segment models may exhibit instability during the calculation process. Analytical models also exhibit deficiencies to varying degrees. For example, while the curve integral model can achieve rigorous modeling of towed streamers, it leads to slightly longer calculation times and struggles to achieve rigorous integration in the elevation direction. The hybrid harmonic function model effectively captures wave characteristics and reduces modeling errors, but its related parameters require further quantitative research. To further reduce and mitigate the risk of misjudging operational hazards such as streamer entanglement or knotting due to calculation with significant modeling errors, this paper proposes a segmented modeling approach based on segmented spline curve fitting. In general, the spline curve model performs better than the polynomial curve model in terms of positioning results in scenarios with high-curvature turns. However, the improvement observed in field experiments is less significant than that observed in simulation experiments. This may be attributed to the following reasons: first, the reference in simulation experiments is the actual coordinate, which serves as a completely accurate reference value, while the error composition in field data is more complex, and the reference values are derived from commercial software calculations. The reference values themselves may contain errors. These residual errors inevitably propagate, causing certain discrepancies in the comparison. Second, both models rely on single-epoch calculations. Some epochs in the actual measurement experiment exhibit missing values and other issues, resulting in varying degrees of jumps, although these deviations are not attributable to the models’ inherent modeling inaccuracies.

Based on the above simulation and actual measurement data test results, the two positioning models are further discussed as follows. First, an analysis of the towed streamer positioning deviation at different distances reveals that the positioning accuracy at the front section, where acoustic observations are lacking, is relatively low. This indicates that dividing the streamer into seven equal-length sections may not match the spatial configuration of the front and rear acoustic networks. In future work, the towed streamer can be divided into sections based on the spatial distribution of sensors on the towed streamer. The number and length of segments will be further studied based on the actual curvature of the streamer, with short segments being deployed in high-curvature areas and longer segments in near-straight sections, enabling adaptive segmentation. Second, the spline curve models for different turning curvatures show varying improvements in accuracy. The spline curve models are better suited for high-curvature turning scenarios, where larger curvatures result in shorter turning times but may increase the risk of streamer entanglement or breakage. Future planning can take into account streamer deployment configurations and prevailing sea conditions to optimize turning radii, thereby balancing modeling errors with optimal estimates. Furthermore, a comparison of streamer shape deviations between the simulation and field experiments reveals that the deviation of the hydrophone along the front-middle section of streamer is greater than that in the middle-rear section during the turning state. This may be due to the limited number of observations in the front-middle section, which is only sparsely covered by the front–rear acoustic network, as well as the different effects of ocean currents on different directions of the towed streamer. Subsequently, the influence of ocean currents on the towed streamer positioning model will be analyzed in combination with shipborne ADCP (Acoustic Doppler Current Profiler) data. In addition, segmented fitting may result in singularity or rank deficiency in the absence of acoustic network constraints, necessitating a balance between the number of segments and calculated efficiency. Finally, cubic polynomials are currently the primary basis functions used. In the future, typical curves such as harmonic functions and B-splines (Basis Spline) will be explored as alternative basis functions for segmented spline fitting of towed streamers.

## 5. Conclusions

This paper presents a spline positioning model that employs cubic polynomials as basis functions and derives constraint equations for the streamer shape at segment junctions. Its performance is evaluated against the polynomial model using both simulated and field data from diverse operational scenarios. The test results show that the positioning accuracy of the two models is essentially the same when the towed streamer is in a straight line. However, during the turning state, the spline model can significantly reduce the modeling error compared with the polynomial curve model. It better describes the true shape of the towed streamer and effectively enhances the positioning accuracy and stability.

## Figures and Tables

**Table 1:** Statistics on mean positioning deviation by epoch (unit: m).

Model	Status	Mean	Standard Deviation	Maximum Value	Minimum Value
Polynomial curve model	Turn	10.86	6.23	30.85	2.54
	Straight	5.09	1.00	7.83	2.49
Spline curve model	Turn	5.74	1.10	10.94	2.88
	Straight	4.66	1.02	7.84	2.77

**Table 2:** Case I: Statistics for mean positioning deviation by epoch (unit: m).

Model	Type	Mean	Standard Deviation	Maximum Value	Minimum Value
Polynomial curve model	Temporal	16.74	6.59	33.81	5.89
	Spatial	16.74	6.28	26.56	5.32
Spline curve model	Temporal	13.39	5.05	28.65	4.82
	Spatial	13.39	5.34	21.94	4.78

**Table 3:** Statistics of mean point position deviation by epoch (unit: m).

Model	Type	Mean	Standard Deviation	Maximum Value	Minimum Value
Polynomial curve model	Temporal	12.34	5.96	37.30	4.78
	Spatial	12.34	2.58	16.81	5.56
Spline curve model	Temporal	8.02	3.40	28.92	2.96
	Spatial	8.02	2.36	12.02	3.14

## Data Availability

The datasets presented in this article are not readily available because the data are part of an ongoing study. Requests to access the datasets should be directed toward Cuilin Kuang.
